# Characterization of New Isolates of *Apricot vein clearing-associated virus* and of a New *Prunus*-Infecting Virus: Evidence for Recombination as a Driving Force in *Betaflexiviridae* Evolution

**DOI:** 10.1371/journal.pone.0129469

**Published:** 2015-06-18

**Authors:** Armelle Marais, Chantal Faure, Eldar Mustafayev, Thierry Candresse

**Affiliations:** 1 INRA, UMR 1332 BFP, Villenave d’Ornon, France; 2 Université de Bordeaux, UMR 1332 BFP, Villenave d’Ornon, France; 3 Genetic Resource Institute of the Azerbaijan National Academy of Sciences, Baku, Azerbaijan; Oklahoma State University, UNITED STATES

## Abstract

Double stranded RNAs from *Prunus* samples gathered from various surveys were analyzed by a deep-sequencing approach. Contig annotations revealed the presence of a potential new viral species in an Azerbaijani almond tree (*Prunus amygdalus*) and its genome sequence was completed. Its genomic organization is similar to that of the recently described *Apricot vein clearing associated virus* (AVCaV) for which two new isolates were also characterized, in a similar fashion, from two Japanese plums (*Prunus salicina*) from a French germplasm collection. The amino acid identity values between the four proteins encoded by the genome of the new virus have identity levels with those of AVCaV which fall clearly outside the species demarcation criteria. The new virus should therefore be considered as a new species for which the name of Caucasus prunus virus (CPrV) has been proposed. Phylogenetic relationships and nucleotide comparisons suggested that together with AVCaV, CPrV could define a new genus (proposed name: Prunevirus) in the family *Betaflexiviridae*. A molecular test targeting both members of the new genus was developed, allowing the detection of additional AVCaV isolates, and therefore extending the known geographical distribution and the host range of AVCaV. Moreover, the phylogenetic trees reconstructed with the amino acid sequences of replicase, movement and coat proteins of representative *Betaflexiviridae* members suggest that *Citrus leaf blotch virus* (CLBV, type member of the genus *Citrivirus*) may have evolved from a recombination event involving a Prunevirus, further highlighting the importance of recombination as a driving force in *Betaflexiviridae* evolution. The sequences reported in the present manuscript have been deposited in the GenBank database under accession numbers KM507061-KM504070.

## Introduction

The family *Betaflexiviridae* is composed of plant viruses with particles of flexuous morphology. Currently, seven genera are recognized in this family, for a total of close to 90 species, including unassigned viruses in the family (http://www.ictvonline.org/virusTaxonomy.asp). During the last five years, a number of viral species belonging to the family *Betaflexiviridae* have been characterized by either classical approaches (e.g. [1–9]) or using high-throughput next generation sequencing (NGS) technologies [10–12]. In the later case, different strategies have been used, targeting different nucleic acid templates (single-stranded or double-stranded RNAs, small interfering RNAs, messenger RNAs, partially or completely purified viral particles) or the sequencing technology (Illumina or 454 pyrosequencing [13–17]). Fruit tree materials have been subjected to a systematic NGS screening of viral infections, in particular to elucidate diseases of still unknown etiology [18]. In this context, the failure to detect viral sequence other than *Little cherry virus 1* (LChV1) in sour cherry sources of the Shirofungen stunt disease led to the suggestion that LChV1 may be responsible for this syndrome [19]. Establishing the association between viral infection and symptomatology is sometimes complicated by mixed viral infections, which are very frequently observed in fruit trees and in other vegetatively propagated plants. For example, a novel Tepovirus infecting *Prunus* species, tentatively named Prunus virus T, was recently characterized following a pyrosequencing analysis of dsRNAs recovered from plum and cherry trees, but it was not possible to associate this new agent with specific symptoms because of its presence in co-infection with common fruit tree viruses [20]. Illumina sequencing of dsRNAs obtained from an apricot tree with vein clearing symptoms recently allowed the identification of a novel unclassified *Betaflexiviridae* member named *Apricot vein clearing associated virus* (AVCaV); however its co-infection with *Plum bark necrosis stem pitting associated virus* (PBNSPaV) similarly hampered the establishment of a causal relationship between AVCaV and the vein clearing symptoms [21].

*Betaflexiviridae* members have genomes which encode a replicase with characteristics of the alphavirus-like superfamily [22–24]. Two genera, *Carlavirus*, *Foveavirus*, and some unassigned members possess a set of three open reading frames (ORFs) collectively known as the triple gene block (TGB), involved in cell-to-cell movement. The remaining genera, *Citrivirus*, *Capillovirus*, *Trichovirus*, *Vitivirus*, and the recently accepted genus *Tepovirus* have a single movement protein (MP) belonging to the "30K-like" superfamily [24–25]. In the genera *Vitivirus* and *Carlavirus*, and in some members of the genus *Trichovirus*, an additional ORF is present 3’ of the coat protein (CP), and encodes an RNA-binding protein with a zinc-ribbon motif, identified as a suppressor of silencing in *Grapevine virus A* [26]. The recently described species AVCaV, proposed as an unassigned species in the family, has a similar genome organization [21]. This novel viral species has only been reported so far from Italy, and a preliminary survey suggested a limited prevalence, with only one variety of apricot and three out of 78 *Prunus* samples found to be infected [21].

In the present study, the complete genome sequence of a novel viral species from an Azerbaijani almond tree, for which the name of Caucasus prunus virus (CPrV) is proposed, was determinated by a deep-sequencing approach. The same strategy provided the genome sequence of two additional isolates of AVCaV, extending our knowledge on the molecular diversity, geographical distribution and host range of this recently discovered virus. Phylogenetic analyses allow definition of a tentative new genus, Prunevirus, composed of CPrV and AVCaV as the type member, and confirm the role of recombination as a driving force in *Betaflexiviridae* evolution.

## Materials and Methods

### Plant samples and virus isolates

The field studies did not involve endangered or protected species and no specific permissions were required for the various locations.

The two Japanese plums (*Prunus salicina*) Pair and 13025 as well as the domestic plum (*Prunus domestica*) 381-07-4 were obtained from the collection held by the Ctifl (Lanxade, France) and were grafted on GF305 peach seedlings under level 3 containment greenhouse conditions. The almond (*Prunus amygdalus*) Aze204 was collected in Arinc, in the mountains of the Nakhchivan area of Azerbaijan. The Japanese apricot (*Prunus mume*) S4 and the apricot (*Prunus armeniaca*) S15 samples were collected during surveys in China. The peach (*Prunus persica*) Iran1 was collected in Iran during a survey in 2005. A number of *Prunus* samples belonging to various species were collected during surveys in Azerbaijan (*P*. *domestica*, *P*. *amygdalus*, *P*. *persica*, *P*. *armeniaca*, and *P*. *avium*), in China (*P*. *mume*, *P*. *armeniaca*, *P*. *sibirica*, and *P*. *persica*), in Kazakhstan (*P*. *armeniaca)*, in Italy (*P*. *armeniaca*, *P*. *avium*, and *P*. *cerasus*), in France (*P*. *domestica* and *P*. *salicina*), and in the Czech Republic (*P*. *cerasus*, *P*. *avium*, *P*. *armeniaca*, and *P*. *domestica)* and were used to assess the prevalence of CPrV and AVCaV. Some *Malus* spp., *Mespilus germanica* and *Cydonia oblonga* samples from Azerbaijan were also tested for virus infection.

### Determination of complete genome sequences by pyrosequencing

Double-stranded RNAs (dsRNAs) were extracted from fresh leaves of GF305 grafted with the 13025 and Pair sources, and from dried leaves of the Aze204 almond. After whole-genome amplification, the amplified fragments were analyzed by 454 pyrosequencing, following the strategy described in Candresse et al. [19]. After demultiplexing, the reads were assembled using the CLC Genomics Workbench 6.5 (http://www.clcbio.com) and annotated by BlastX and BlastN comparison with GenBank, using a 10^−3^ e-value cut-off. Mapping of contigs on the genome of AVCaV (for the 13025 and Pair sources) or of *Citrus leaf blotch virus* (CLBV) (for the Aze204 source) used as references allowed the scaffolding and ordering of contigs for each viral isolate. In order to complete the genomic sequences, the gaps between the contigs generated from the pyrosequencing data as well as regions for which sequence confirmation was sought were amplified from total nucleic acids (TNA) extracted from the 13025, Pair and Aze204 sources with primers designed from the sequence of the contigs (S1 Table) in two-step RT-PCR procedures, as described by Marais et al. [27]. The 5' end of each viral genome were determined using a Random Amplification of cDNA Ends (RACE) strategy and internal primers designed from the contigs (S1 Table), following the manufacturer's instructions (Takara Bio Europe/Clontech^©^, Saint-Germain-en-Laye, France). When needed, a nested amplification was performed to increase the amplification signal. The 3’ genomic regions were amplified using forward internal primers designed from the contigs and the LD-prime primer in long-distance PCR amplification reactions following the protocol described in Youssef et al. [2] (S1 Table). All amplification products were sequenced on both strands (GATC Biotech AG, Mulhouse, France), either directly or after a cloning step into the pGEM-T Easy vector (Promega, Charbonnières-Les Bains, France). The sequences obtained were finally assembled with the initial contigs to generate the complete genomic viral sequences.

### Determination of the partial genomic sequence of the AVCaV Iran1 isolate

TNA were extracted from *P*. *persica* Iran1 leaves according to the protocol 2 described by Foissac et al. [28] and submitted to the Polyvalent Degenerated Oligonucleotide (PDO) nested RT-PCR as described by Foissac et al. [28], targeting the active site of the viral polymerase. A primer (IF1: 5' GTGTGTTGAGTCTGATTACGAAG 3') was designed from the nucleotide sequence of the PDO fragment and used, together with LD-polyT and LD-prime primers (S1 Table) to amplify the region between the PDO fragment and the polyA tail at the 3' end of the genome using Long-Distance (LD) PCR. The amplified fragment of 2.8 kbp was cloned into the pGEM-T Easy vector (Promega) and sequenced on both strands (GATC Biotech AG).

### Total nucleic acids extraction and detection of AVCaV and the new CPrV by RT-PCR

TNA were extracted from dried or fresh leaf samples as described above. Specific detection of AVCaV was performed following the two-step RT-PCR assay described by Elbeaino et al. [21]. This assay allows the amplification of a short fragment (*ca*. 330 nt) of the replicase gene using primers VC37657s and VC28239a. Two primer pairs were designed in the present study to allow the simultaneous detection of AVCaV and CPrV isolates. One primer pair (NB-F1i: 5' ATGYTIGTIMGIAARYTIGARATHCARGA 3' and NB-R1: 5' GAACTKACYAAAACTGGCAARGTCTCTGA 3') allows the amplification of a 274-nt long fragment in the nucleic acid binding protein gene (NB). The other one (Pol-F1i: 5' CARYTITGYACIAARTAYGARAARCARTAYGT 3' and Pol-R1: 5' CCRATWGCSTTTGCTGGGGATGAYATGTG 3') was designed to amplify a fragment of 499 nt in the polymerase gene. A two-step RT-PCR assay was applied. TNA (5 μl) were submitted to a reverse transcription following the protocol described by Marais et al. [29]. The cDNA (5 μl) was then amplified in a 50-μl volume containing 10 mM Tris-HCl (pH8.8), 1.5 mM MgCl_2_, 50 mM KCl, 250 μM dNTPs, primers at 1 μM each, and 1 U of DyNAzyme II DNA polymerase (Finnzymes/Fisher Scientific, Illkirch, France) and 40 cycles were applied, each of 95°C for 30 sec, 48°C (NB-F1i/R1) or 42°C (Pol-F1i/R1) for 30 sec, and 72°C for 30 sec, followed by a final extension step of 10 min at 72°C. The amplified fragments were visualized under UV light after non-denaturating electrophoresis on agarose gel and ethidium bromide staining. The amplicons were sequenced (GATC Biotech AG) either directly, or after a cloning step into pGEM-T Easy vector (Promega).

### Sequence and phylogenetic analyses

Manipulations of 454 pyrosequencing sequence data were performed as described by Candresse et al. [19] using the CLC Genomics Workbench 6.5. Phylogenetic and molecular analyses were conducted using MEGA version 6 [30]. Phylogenetic trees were reconstructed using the neighbor-joining technique with strict nucleotide or amino acid distances and randomized bootstrapping for the evaluation of branching validity. Genetic distances (p-distances calculated on nucleotide or amino acid identity) were calculated using MEGA version 6.

## Results

### Pyrosequencing of dsRNAs extracted from the Pair and 13025 Japanese plum sources and from the Aze204 almond sample

After demultiplexing and quality trimming of 454 pyrosequencing data, a total of 29,538 reads were obtained for the Pair source, 16,750 reads for the 13025 source, and 18,418 reads for the Aze204 sample. These sequences were then treated as previously described [19]. Blast comparisons following *de novo* assembly of contigs showed that in the Pair source three viruses were present in co-infection, namely PBNSPaV (two isolates, collectively representing 60.9% of the reads, [29]), *Prunus necrotic ring spot virus* (PNRSV, 1.3% of the reads), and an isolate of AVCaV, representing 10.8% of the reads [29]. For the latter virus, a long contig of 8,120 nucleotides (nt) was obtained, spanning the AVCaV genome from nt 205 to nt 8,325.

In the 13025 Japanese plum sample, Blast comparisons allowed identification of two viruses: PBNSPaV (two distinct isolates, 43% of the reads) and an isolate of AVCaV, representing 2% of the reads. Seven contigs identified as belonging to AVCaV were manually assembled by mapping against the reference AVCaV genome, thus creating a scaffold of 6,141 nt missing the 5' and 3' genome ends and containing six internal gaps. The genomic sequences of the Pair and 13025 AVCaV isolates were completed by targeted sequencing as described in the Materials and Methods section. The assembled complete genome sequences were deposited in the GenBank database under accession numbers KM507062 and KM507063, respectively.

For the Aze204 almond sample, only a few contigs could be identified as having a possible viral origin by BlastN and BlastX analyses. Five contigs representing 0.09% of the reads and totaling 1,853 nt were thus shown to have weak sequence similarities to various members of family *Betaflexiviridae* such as AVCaV, CLBV, *Apple chlorotic leaf spot virus* (ACLSV), or *Asian prunus virus 1* (APV1), depending on the contig considered. Assuming that all contigs belonged to the same agent, a scaffold was constructed by alignment with the CLBV sequence, which spanned the CLBV genome from positions 403 to 6,820, with four internal gaps. Primers were designed from the contig sequences and amplifications using Aze204 source TNA were performed in order to complete and validate the sequence of this tentative scaffold. As in previous efforts [19,20,29], essentially no differences were observed between the initially assembled scaffold and the resequenced regions, thus validating both the initial contigs and the tentative scaffolding. The assembled sequence was deposited under accession number KM507061 in the GenBank database.

### Partial sequencing of the AVCaV isolate present in the Iran1 *Prunus persica* source

TNA extracts from the Iran1 *P*. *persica* source were submitted to a PDO nested RT-PCR which permits the detection of *Trichovirus*, *Foveavirus*, and *Capillovirus* members as well as of some unassigned *Betaflexiviridae* members [28]. In Blast analyses, the 310-nt long fragment obtained showed strong nucleotide identity (92%) with the corresponding fragment of AVCaV [21]. Using a forward primer located in the PDO fragment and LD-polyT primer targeting the polyA tail of the genome, a 2,883-nt long fragment was amplified, cloned and sequenced on both strands by primer-walking. The assembled sequence of 3,037 nt was deposited under Accession number KM507070 in the GenBank database.

### Comparison of AVCaV isolates genomic sequences

**Fig 1:**
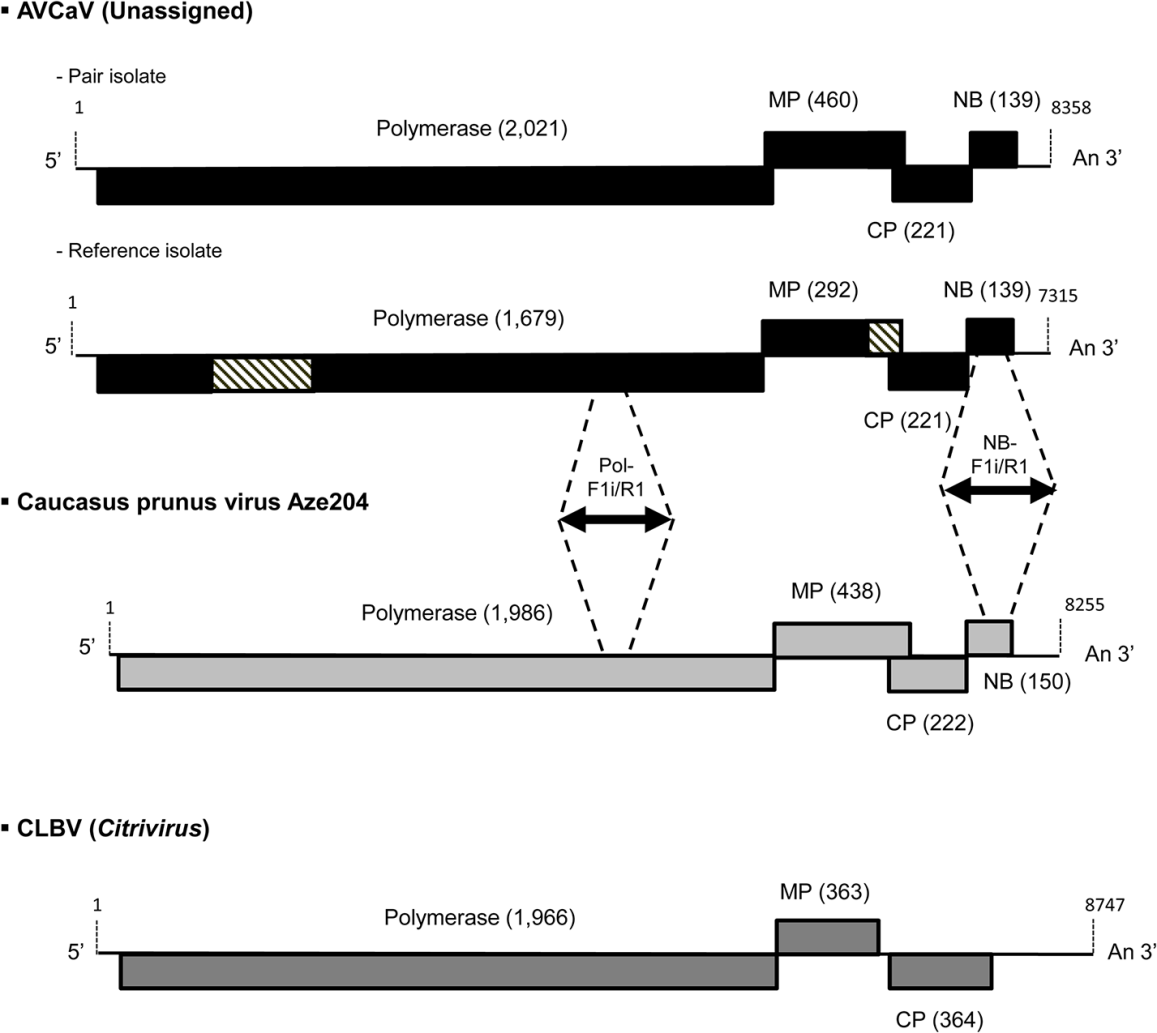
Schematic representation of the genomes of *Apricot vein clearing associated virus* (AVCaV, reference isolate and Pair isolate), Caucasus prunus virus (Aze204 agent) and *Citrus leaf blotch virus* (CLBV). The size in amino acids of the encoded proteins is indicated between brackets. The four genomes are aligned on the basis of the C-ter of the polymerase. The hatched boxes represent parts of the Pol and MP genes which are either absent or not expressed, respectively, in the AVCaV reference isolate but present and expressed in the Iran1, Pair and 13025 isolates. The black arrows represent the amplified fragments using the two primer pairs Pol-F1/R1 and NB-F1/R1. MP, Movement protein; CP, Coat protein; NB, Nucleic acid binding protein.

Complete genome sequences of three AVCaV isolates and one partial (3,037 nt 3' terminal) are now available. Both complete AVCaV sequences determined here are 8,358 nt long, significantly longer than the reference isolate (7,315 nt). Their overall genomic organization and gene order are conserved as all encode four proteins from 5' to 3' (Fig 1): a replication-associated protein (Pol), a movement protein (MP), a coat protein (CP) and a nucleic acid binding protein (NB). The CP is the only protein conserved in size (221 amino acids [aa]) between the four AVCaV isolates (S2 Table). The Pol of the 13025 and Pair isolates contains an additional 342 aa inserted at aa position 405 of the Pol reference isolate, so that the size of their protein is 2,021 aa instead of 1,679 for the previously sequenced isolate [21]. The five domains methyltransferase (MeT), AlkB, endopeptidase, helicase and RNA-dependent RNA polymerase are conserved, with the large indel positioned between the MeT and AlkB domains, in the hypervariable part of the *Betaflexiviridae* Pol protein. Interestingly, in the 13025 and Pair isolates, the indel is bordered by the duplicated heptanucleotide sequence GCAACTT. The MP of the reference isolate is also significantly shorter (292 aa) due to a frameshift mutation leading to premature MP ORF termination as compared to the other three isolates, which have a MP of 460 aa. Three of the four AVCaV isolates encode a NB of 139 aa, but that of the Iran1 isolate is 23 aa longer due to a point mutation (T to C) at nucleotide 419 in the NB gene, which suppresses the first stop codon at the end of the ORF. The large indel in the Pol gene and the mutations affecting the length of the various ORFs were all confirmed by targeted sequencing of the relevant isolates. The 5' non coding region (NCR) of the Pair and 13025 isolates is 78 nt long, five nt longer than that of the reference isolate, due to indels. The 3' NCR is also variable is size, being 152 nt long in the Pair and 13025 isolates (13 nt longer at the 3' end than in the reference isolate) but only 84 nt long in the Iran1 isolate, due to the extension of the NB gene to the second in frame stop codon. Overall, and when not taking into account the large indel in the Pol gene, the three complete genomes show a very high level of nucleotide identity (96%-96.8%). As shown in S2 Table, the same applies when considering the different genome regions (and the fourth, partially sequenced isolate), with the exception of the 5' NCR, which is highly divergent in the reference isolate and shows only about 58% identity with that of the other two isolates.

### Genome organization of Aze204 agent and determination of its phylogenetic affinities

**Fig 2:**
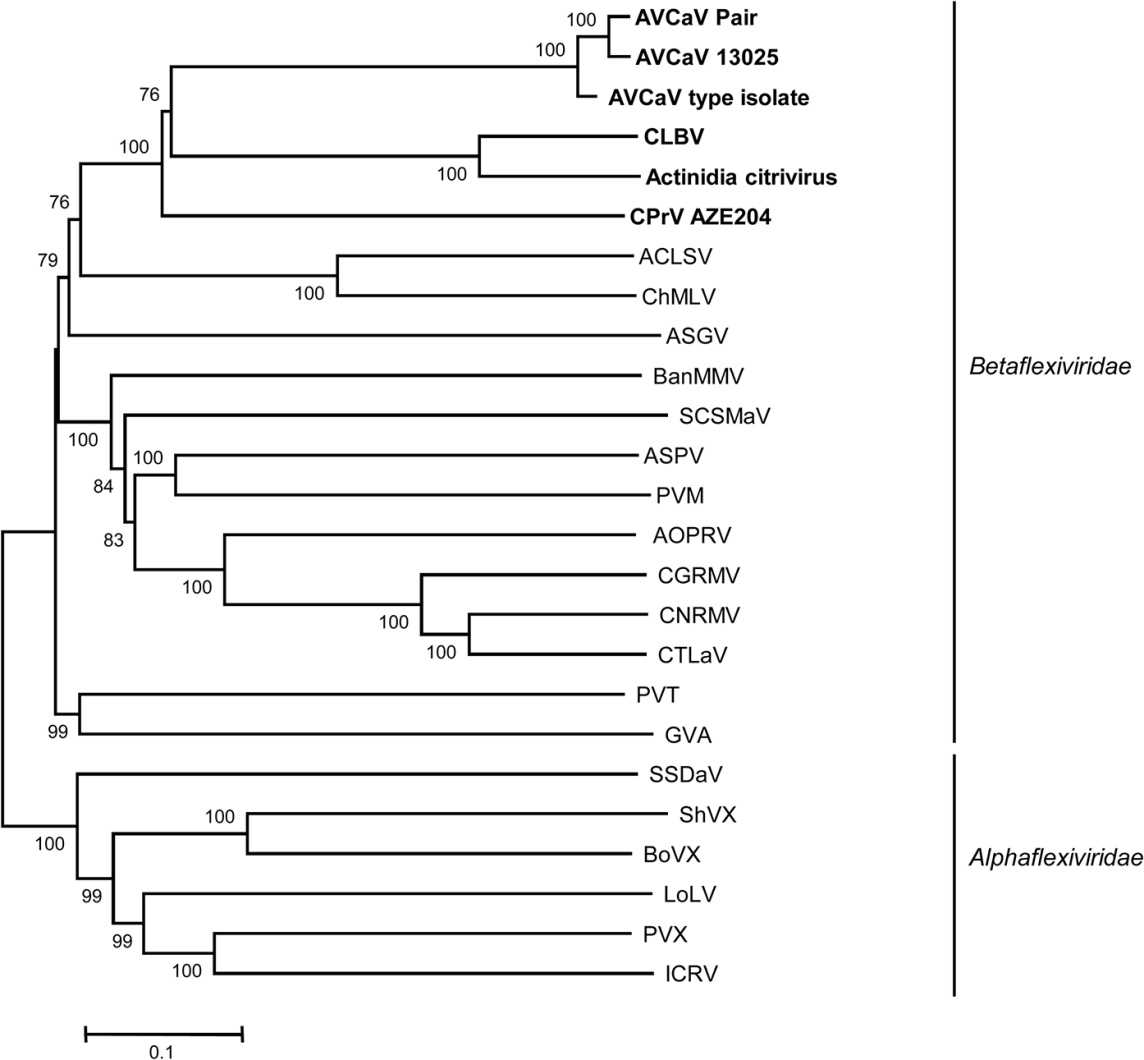
Phylogenetic analysis of the polymerase amino acid sequences of representative species in the *Alphaflexiviridae* and *Betaflexiviridae* families. The abbreviations of virus names are given in S3 Table. The families to which each virus belongs are indicated at the right. The isolates of AVCaV, CLBV and CPr VAze204 are indicated in bold. The trees were constructed by the neighbour-joining method from strict amino acid identity distances and the statistical significance of branches was evaluated by bootstrap analysis (1,000 replicates). Only bootstrap values higher than 70% are indicated. The scale bars represent 10% amino acid divergence.

**Fig 3:**
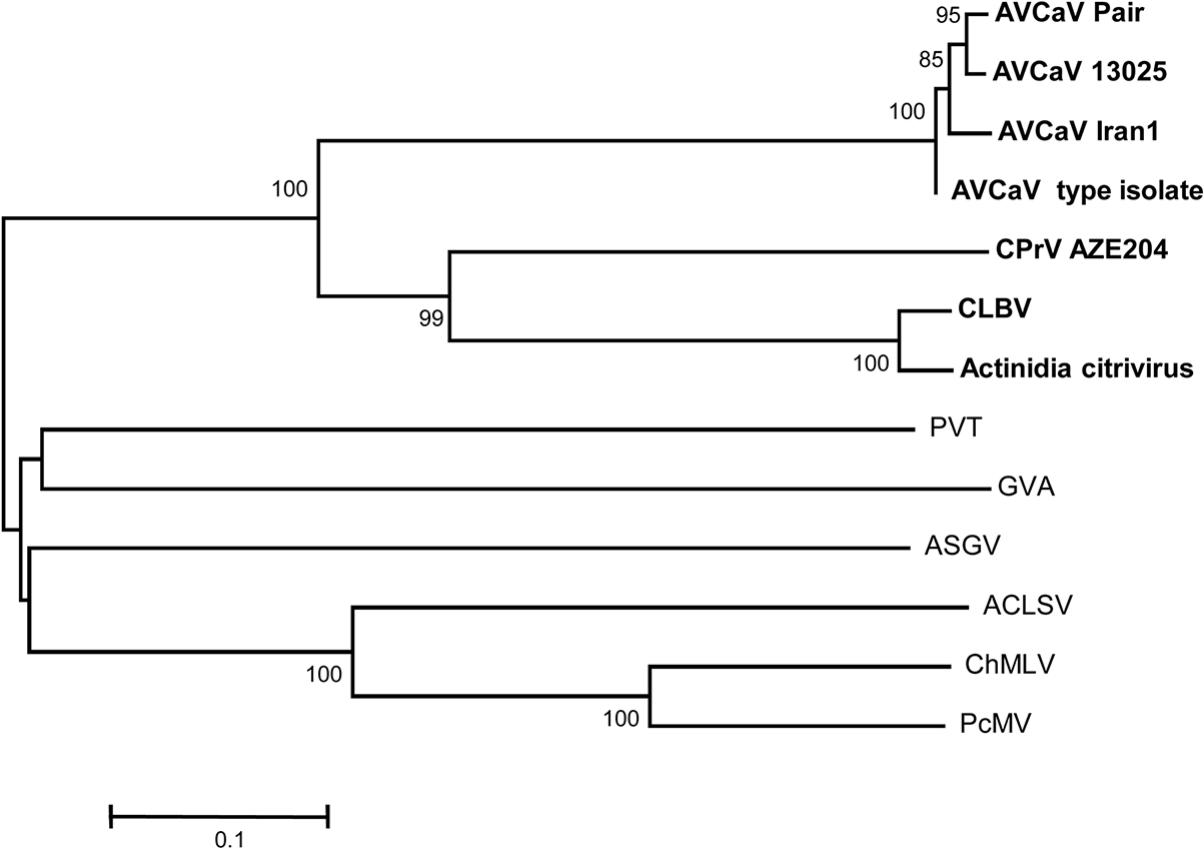
Phylogenetic analysis of the movement protein amino acid sequences of representative species in the family *Betaflexiviridae*. The abbreviations of virus names are given in S3 Table. The isolates of AVCaV, CLBV and CPrV Aze204 are indicated in bold. The trees were constructed by the neighbour-joining method from strict amino acid identity distances and the statistical significance of branches was evaluated by bootstrap analysis (1,000 replicates). Only bootstrap values higher than 70% are indicated. The scale bars represent 10% amino acid divergence.

**Fig 4:**
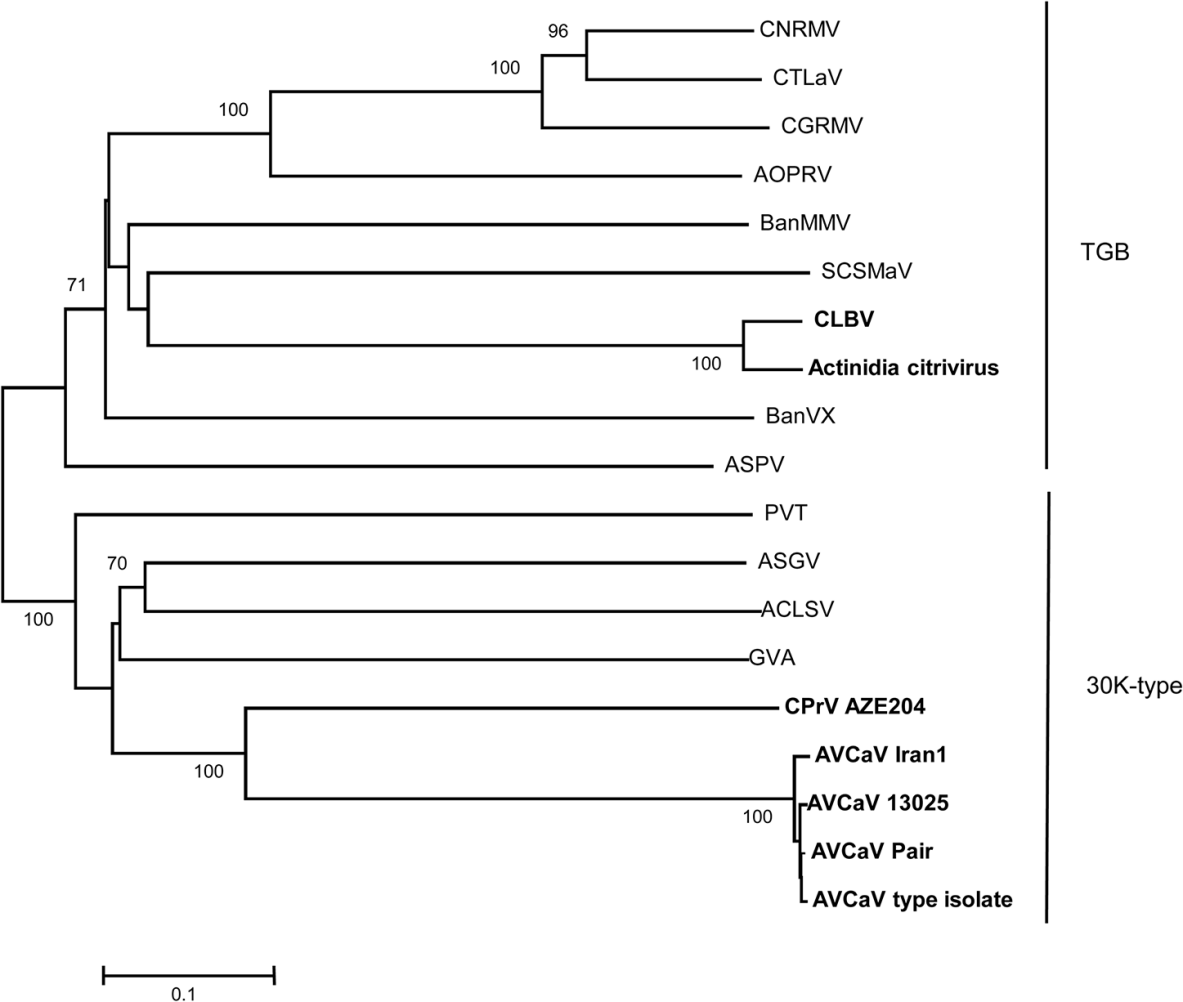
Phylogenetic analysis of the coat protein amino acid sequences of representative species in the family *Betaflexiviridae*. The abbreviations of virus names are given in S3 Table. The type of the movement protein (30K-type or Triple Gene Block, TGB) to which each virus belongs are indicated at the right. The isolates of AVCaV, CLBV and CPrV Aze204 are indicated in bold. The trees were constructed by the neighbour-joining method from strict amino acid identity distances and the statistical significance of branches was evaluated by bootstrap analysis (1,000 replicates). Only bootstrap values higher than 70% are indicated. The scale bars represent 10% amino acid divergence.

The genome of the virus found in the Aze204 source has a size of 8,255 nt excluding the polyA tail. The genome structure is similar to that of some members of family *Betaflexiviridae*, namely *Cherry mottle leaf virus* and *Peach mosaic virus* (both members of the genus *Trichovirus*), and AVCaV, with relatively short NCRs (65 nt at 5' and 152 nt at 3') and four encoded proteins: a replication-associated protein (Pol, 1,986 aa), a 30K-type movement protein (MP, 438 aa), a coat protein (CP, 222 aa), and a nucleic acid binding protein (NB, 150 aa) (Fig 1). In addition to the similar genomic structure, the sizes of the encoded proteins are similar to those of AVCaV, especially when considering the two AVCaV isolates characterized in the present work (see above). Blast analyses failed to reveal any closely related agent, AVCaV being detected as the closest but still quite distant relative (results not shown). Phylogenetic analysis of the sequences of the encoded proteins show that Aze204 agent and AVCaV cluster together with highly significant bootstrap values (100%) whatever protein is considered (Figs 2–4 and S1 Fig). The same clustering is obtained using whole genome sequences (data not shown). Comparison with sequenced *Betaflexiviridae* members showed that whatever ORF is considered, only distant relationships and identity levels are observed, with the sole exception of the NB ORF, which shows 73% amino acid identity with that of AVCaV (Table 1). As observed in the Blast analyses, the closest relative of the new agent is consistently AVCaV, which shows only 47.1–51.6% nt identity (36.5–44% aa identity) for the CP, MP and Pol genes (Table 1). Phylogenetic analyses at the whole genome level and for the Pol and MP genes also reveal affinities between AVCaV, the Aze204 agent and CLBV, the type member of the genus *Citrivirus* [31]. The Pol gene of CLBV shows 49% nucleotide identity (40.7% amino acid identity) with those of AVCaV and of the Aze204 agent, and even higher values are observed when comparing the MPs (Table 1). However, the CLBV CP has no detectable phylogenetic affinities with those of AVCaV and of the Aze204 agent (Fig 4) and very low nucleotide (30.4%) or amino acid (11.2%) identity (Table 1). In fact, the CLBV CP has affinities with those of *Betaflexiviridae* members with a triple gene block (TGB in Fig 4).

**Table 1:** Percentage of identity of genes and encoded proteins of the new agent identified in the Aze204 *Prunus* source with the corresponding genes and proteins of *Betaflexiviridae* members with a 30K-type movement protein.

Virus species	Pol	MP	CP	NB
AVCaV	51.6 *(44)*	50.2 *(42*.*1)*	47.1 *(36*.*5)*	68.3 *(73)*
CLBV	49 *(40*.*7)*	56.7 *(53)*	30.4 *(11*.*2)*	na^a^
PVT	42.9 *(29*.*2)*	29.9 *(13*.*4)*	38.7 *(21*.*6)*	na^a^
ASGV	40.2 *(26*.*5)*	30.9 *(13*.*5)*	36.7 *(25*.*9)*	na^a^
GVA	40.1 *(27)*	30.2 *(9*.*2)*	38.3 *(24*.*9)*	34.4 *(11*.*1)*
ChMLV	40.9 *(26*.*4)*	33.4 *(13*.*2)*	41.1 *(30*.*4)*	32.4 *(13*.*6)*
ACLSV	40.8 *(26*.*8)*	31.1 *(10*.*2)*	39.1 *(24*.*6)*	na^a^

The amino acid identity percentages are indicated in italics between brackets. Pol, Polymerase; MP, Movement protein; CP, Coat protein; NB, Nucleic acid binding protein. The abbreviations of virus names are given in S3 Table.

^a^ not applicable

### Development of a broad-spectrum detection test for AVCaV and the new Aze204 agent

The comparison of the three complete genome sequences of AVCaV (Pair, 13025 and the reference isolate) in addition to the partial sequence of Iran1 isolate and the complete sequence of the new agent from the Aze204 source allowed the identification of several conserved motifs in the deduced proteins. Two conserved regions were selected, one in the Pol and the other in the NB. Low degeneracy inosine-containing primers targeting these two regions were designed. As presented in S2–S3 Figs, the two viral species (AVCaV and the Aze204 agent) were efficiently and reproducibly detected in Japanese plums (lanes 4 and 7) and in almond (lane 2), yielding the expected 274- or 499-bp PCR products. The detection test targeting the NB gene consistently gave stronger amplification signals than the one targeting the Pol gene.

To assess the incidence of both viruses in various *Prunus* accessions, 412 samples gathered during several surveys were submitted to the polyvalent RT-PCR assays. No additional sample was found to be infected by the new virus. In contrast, three additional samples were found to be infected with AVCaV (S2–S3 Figs lanes 3, 5, and 6), enlarging the natural host range of this virus to *P*. *mume* (sample S4), and to *P*. *domestica* (sample 381-07-4), and its geographical distribution to China (samples S4 and S15) (S4 Table). Sequencing of the amplified fragments confirmed the specificity of the detection assays. The unrooted neighbor-joining trees constructed using the partial sequences of the NB gene or of the Pol gene showed that all identified AVCaV isolates appear to form a relatively tight cluster, with the exception of the Iran1 isolate, which is more divergent (data not shown). The intragroup nucleotide diversity in the cluster of closely related isolates is in the same range for both genomic regions (1.5% and 1.4% nucleotide divergence in the NB and Pol regions, respectively), whereas the nucleotide divergence between the Iran1 isolate and the major group reaches 5.4% in the NB region and 7.4% in the Pol region.

## Discussion

In the present study, the high-throughput sequencing of double stranded RNAs extracted from various fruit tree samples allowed the detection and the efficient sequencing of the complete genome of two isolates of the newly described AVCaV and the identification of a novel virus belonging to the family *Betaflexiviridae*. These results further illustrate the power of the NGS (Next Generation Sequencing) based approaches to identify and characterize known or novel fruit tree viruses [12,19–21,29,32,33].

The comparison of the two complete and of the partial AVCaV genomic sequences determined here with the type isolate from Italy (NC 023295, [21]) show the Italian isolate to have several unique features, including the large, 1,029 nt deletion in the hypervariable region of the Pol gene and a 168 aa-shorter MP caused by a frameshift mutation. The impact of these mutations on the biology or even on the infectivity of the Italian isolate remain to be evaluated. The existence of defective RNAs (D-RNAs) has been reported before in the family *Betaflexiviridae* [34] but these molecules had deletions of 1.2–1.5 kb, spanning several 3’ genome genes. The fact that these deletions were bordered by short 2 to 4 bp duplications [34] may constitute a parallel to the observation that the Italian AVCaV isolate deletion is bordered by the duplicated heptanucleotide GCAACTT. Nevertheless, all isolates show high genome identity values despite their very different origins, suggesting a narrow genetic diversity of AVCaV, which will need to be confirmed by further studies. AVCaV was first discovered in an apricot tree from Italy and a preliminary survey indicated a limited spread of the virus in nature [21]. The present work allowed estimation of a ca. 1.4% incidence of AVCaV in a large *Prunus* collection and to enlarge its known geographical distribution to three further countries (France, China, and Iran), and its natural host range to several *Prunus* species: *P*. *salicina*, *P*. *persica*, *P*. *mume*, and *P*. *domestica*. However, as in the work of Elbeaino et al. [21], due to mixed viral infections in all the seven identified AVCaV-positive trees, it is still difficult to associate specific symptoms with AVCaV infection. All identified host plants were simultaneous infected by common fruit tree viruses (S4 Table), such as *Plum pox virus* (*Potyvirus*), *Cherry virus A* (*Capillovirus*), PBNSPaV, or PNRSV, as well as by a putative new *Trichovirus* species related to PcMV and ChMLV [29], so that it is not possible to associate the symptoms observed on the leaves of the various trees, such as marbling, chlorosis, or reddening with AVCaV presence.

The genomic organization, sequence comparisons and phylogenetic analyses show that the viral agent identified in the Azerbaijani almond plant Aze204, has its closest affinities with AVCaV. However, the nucleotide or encoded amino acid sequence identities for their CP and Polymerase genes clearly fall below the 72% (nt identity) or 80% (aa identity) values used as species demarcation criteria in the family *Betaflexiviridae* [24]. The new agent should therefore be considered as a new and distinct species, for which the name of Caucasus prunus virus (CPrV) is proposed. However, the identical genomic organization of AVCaV and CPrV and their nucleotide identities in the CP and Pol genes (47.1% and 51.6%, respectively), above the 45% genus demarcation criteria in the family *Betaflexiviridae* strongly support the idea that both viruses should be assigned to the same genus.

As reported by Martelli et al. [35], most flexiviruses show a strong correlation between the phylogenetic affinities of their CP and the type of MP encoded by the genome. CLBV is so far the only exception to this rule. In the present work, clear affinities were observed between the Pol and MP genes of AVCaV/CPrV and CLBV, with identity values that are above the 45% genus discrimination criterion (Table 1). On the other hand, their 3’ genome parts are strikingly different, showing extremely low identity levels (Table 1), clearly different phylogenetic affinities for the CPs and lack of a NB gene for CLBV. Taken together, these observations are highly suggestive of an ancestral recombination event, with all three agents having inherited the same ancestral 5’ genome part (polymerase and MP) but 3’ genome parts of different origins (30K-type MP *Betaflexiviridae* CP plus NB for AVCaV and CPrV; TGB-*Betaflexiviridae* CP for CLBV). Moreover, the clustering of CLBV with Actinidia citrivirus [7] whatever the protein considered (Figs 2–4) strongly suggests that the genus *Citrivirus* should result from a single recombination event followed by diversification.

There already exist cases in the family *Betaflexiviridae* where members of the same genus differ by presence or absence of a NB ORF (*Trichovirus* and *Carlavirus* genera). However, the agents concerned share clear phylogenetic affinities in all three other genome ORFs (polymerase, MP, CP) and have Pol and CP genes identity levels that fall within the genus demarcation criteria. CLBV and AVCaV/CPrV not only have different genomic organization when it comes to possessing or not a NB ORF but have also strikingly different CP phylogenetic affinities, in the TGB- and 30K-type MP *Betaflexiviridae* members, respectively. Therefore, we propose that AVCaV and CPrV should not be included together with CLBV in the genus *Citrivirus*. Given their original genome organization and high divergence from any other member of the family, we suggest that AVCaV and CPrV should rather be integrated in a newly created genus, for which the name Prunevirus is proposed.

As reviewed by Sztuba-Solinska et al. [36], recombination is a relatively common process in plant RNA virus evolution, even if the rates of recombination can dramatically vary between viruses [37]. The recombination frequency is reported to be important in retroviruses and in positive single stranded RNA viruses, in particular in families *Coronaviridae*, *Bromoviridae* and *Potyviridae* [37,38]. Few studies dealing with recombination in the family *Betaflexiviridae* have been published [39–43]. Martelli et al. [35] postulated a common ancestor for the families *Betaflexiviridae*, *Alphaflexiviridae* and *Tymoviridae*, and subsequent recombination events and gene loss to generate various plant and fungal viruses. The role of such reassortments of viral genomes in the acquisition of adaptation to new hosts has also been suggested in several studies [44–46]. The detailed analysis of the genomes of AVCaV, CPrV and CLBV performed here further support the concept that recombination is likely to have played a major role in the macroevolution of members of the family *Betaflexiviridae*.

The identification of conserved domains in the AVCaV and CPrV genomes allowed the design of two primer pairs targeting conserved regions between the four AVCaV isolates and CPrV. Both primer pairs were used, with similar results, for a systematic search by RT-PCR of AVCaV and CPrV, even if the one targeting the NB gene seems to be more sensitive. The ability of these molecular assays to detect more broadly members of the proposed genus Prunevirus should be evaluated as soon as other viral agents belonging to this new genus are characterized. As for AVCaV, the biological information on CPrV is extremely scarce. No additional sample infected by CPrV was detected among the collection of *Prunus* tested, suggesting a limited spread of this agent. The symptoms shown by the CPrV-infected Aze204 almond tree were chlorotic spots located along the veins and weak reddening of young leaves. The failure to detect any other viral agent in the Aze204 source by the deep sequencing approach used suggests that CPrV is likely to be responsible for those symptoms. However, the symptoms could be also due to another agent (DNA virus, viroid) falling outside of the detection range of the dsRNA sequencing strategy used. Further investigations will therefore be necessary to unambiguously associate CPrV with the symptoms observed.

## Supporting Information

S1 Fig Unrooted phylogenetic tree calculated from amino acid sequences of the Nucleic acid binding (NB) proteins of representative members of the family *Betaflexiviridae*. The abbreviations of virus names are given in S3 Table. The tree was reconstructed by the neighbour-joining method from strict amino acid identity distances and the statistical significance of branches was evaluated by bootstrap analysis (1,000 replicates). Only bootstrap values higher than 70% are indicated. The scale bar represents 10% amino acid divergence. The four isolates of AVCaV and CPrV Aze204 are indicated in bold.
(PDF)

S2 Fig Detection of AVCaV and the new agent Aze204 using broad-spectrum reverse-transcription polymerase chain reaction assay based on the degenerate primers NB-F1i/R1. The size of the amplified fragments is indicated near the arrows. Samples analyzed were: lane 1, negative control; lane 2, Aze204; lane 3, S15; lane 4, Pair; lane 5, 381-07-4; lane 6, S4; lane 7, 13025; lane L, 1 kb Plus DNA ladder (Life Technologies /ThermoFisherScientific, Illkirch France). A precise description of the *Prunus* sources used is provided in S4 Table.
(PDF)

S3 Fig Detection of AVCaV and the new agent Aze204 using broad-spectrum reverse-transcription polymerase chain reaction assay based on the degenerate primers Pol-F1i/R1. The size of the amplified fragments is indicated near the arrows. Samples analyzed were: lane 1, negative control; lane 2, Aze204; lane 3, S15; lane 4, Pair; lane 5, 381-07-4; lane 6, S4; lane 7, 13025; lane L, 1 kb Plus DNA ladder (Life Technologies /ThermoFisherScientific). A precise description of the *Prunus* sources used is provided in S4 Table.
(PDF)

S1 Table List of primers used to amplify and sequence internal gaps, terminal regions and regions of low coverage of 454 pyrosequencing scaffolds for AVCaV or CPrV isolates identified in the Aze204, Pair and 13025 *Prunus* sources. (DOCX)

S2 Table List of virus species used for the phylogenetic analyses. (DOCX)

S3 Table Percentages of identity between various regions of AVCaV isolate Pair and corresponding regions of AVCaV reference isolate (NC 023295), AVCaV Iran1, and AVCaV 13025. (DOCX)

S4 Table List of *Prunus* sources found infected by a virus belonging to the proposed genus Prunevirus. (DOCX)
