# Supplementary figures and images for: Characterization of New Isolates of Apricot vein clearing-associated virus and of a New Prunus-Infecting Virus: Evidence for Recombination as a Driving Force in Betaflexiviridae Evolution
Source: PLoS One. 2015 Jun 18;10(6):e0129469. doi: 10.1371/journal.pone.0129469 (PMC4472227; doi:10.1371/journal.pone.0129469)

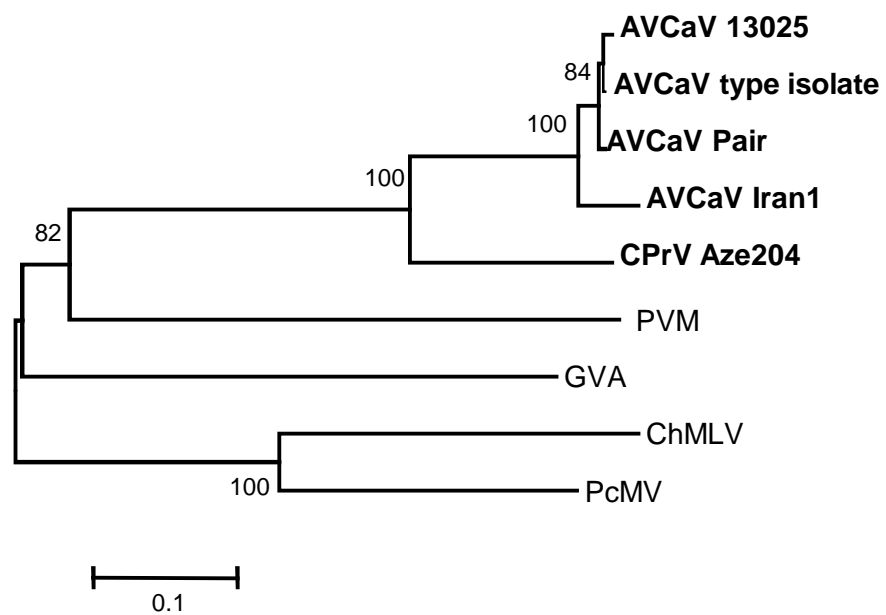

Supplement: S1 Fig — The abbreviations of virus names are given in S3 Table. The tree was reconstructed by the neighbour-joining method from strict amino acid identity distances and the statistical significance of branches was evaluated by bootstrap analysis (1,000 replicates). Only bootstrap values higher than 70% are indicated. The scale bar represents 10% amino acid divergence. The four isolates of AVCaV and CPrV Aze204 are indicated in bold. (PDF) [file pone.0129469.s001.pdf]

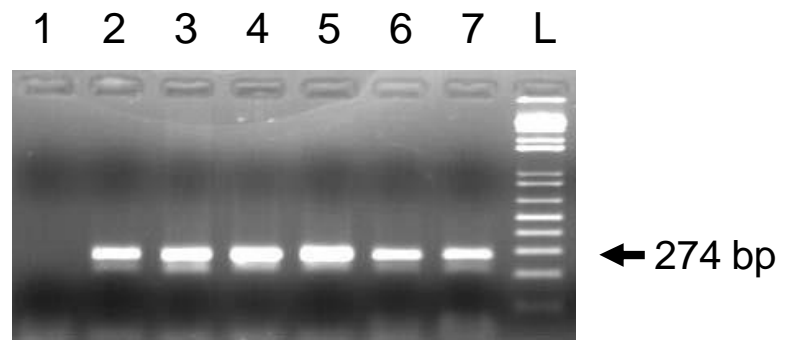

Supplement: S2 Fig — The size of the amplified fragments is indicated near the arrows. Samples analyzed were: lane 1, negative control; lane 2, Aze204; lane 3, S15; lane 4, Pair; lane 5, 381-07-4; lane 6, S4; lane 7, 13025; lane L, 1 kb Plus DNA ladder (Life Technologies /ThermoFisherScientific, Illkirch France). A precise description of the Prunus sources used is provided in S4 Table. (PDF) [file pone.0129469.s002.pdf]

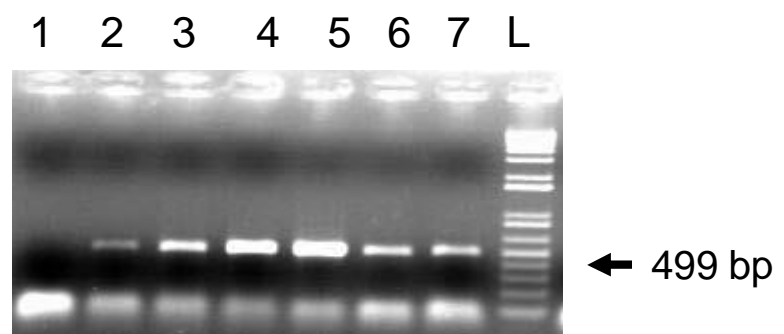

Supplement: S3 Fig — The size of the amplified fragments is indicated near the arrows. Samples analyzed were: lane 1, negative control; lane 2, Aze204; lane 3, S15; lane 4, Pair; lane 5, 381-07-4; lane 6, S4; lane 7, 13025; lane L, 1 kb Plus DNA ladder (Life Technologies /ThermoFisherScientific). A precise description of the Prunus sources used is provided in S4 Table. (PDF) [file pone.0129469.s003.pdf]
