# Supplementary material for: Characterization of New Isolates of Apricot vein clearing-associated virus and of a New Prunus-Infecting Virus: Evidence for Recombination as a Driving Force in Betaflexiviridae Evolution
Source: PLoS One. 2015 Jun 18;10(6):e0129469. doi: 10.1371/journal.pone.0129469 (PMC4472227; doi:10.1371/journal.pone.0129469)
Supplement: S1 Table — (DOCX) [file pone.0129469.s004.docx]

**S1 Table. List of primers used to amplify and sequence internal gaps, terminal regions and regions of low coverage of 454 pyrosequencing scaffolds for AVCaV or CPrV isolates identified in the Aze204, Pair and 13025 *Prunus* sources.**

| **Primer name** | **Sequence 5'-3'** | **5' position^a^** |
| --- | --- | --- |
| **Aze204** |  |  |
| Aze204-F1 | GTCTTTCCACCTGAGCTTCT | 663 |
| Aze204-R1 | GGTTATGCCCATAATCAATAGA | 2694 |
| Aze204-F2 | GGGTAGTATTGGAATGCATAGGGA | 2798 |
| Aze204-R2 | TCAGAGTCTGAGTGGTACCTAG | 3973 |
| Aze204-F3 | CTCATTAGGGCTAAGGAATAC | 4716 |
| Aze204-R3 | TCCATACTCCAGTCAATGTCAG | 5128 |
| Aze204-F4 | TGAAGAAGGAGAGAAGGTGGAG | 5357 |
| Aze204-R4 | CTGGAAAGATTCCATCCACAGAACAT | 5770 |
| LD-Aze204^b^ | ACGTAGCACGCCCAAATGGAGCTTG | 6609 |
| Race-Aze204^c^ | GTGCCAGCCGAAGTGTGGATGTAACTAGC | 844 |
| **13025** |  |  |
| LD-Flex-F1^b,d^ | GAGGAGAGCTAAACGTCTTGG | 7957 |
| Race-Flex1^c,d^ | ACCCATAAGCTGCTTGAGCTTAGCC | 1114 |
| NRace-Flex1^c,d^ | GGAAGGATGTCTGGGGCCTCACGAC | 543 |
| 13025-F2 | ACATTCCTCTTCAATACTTTGGC | 5662 |
| 13025-R2 | AATTTAGATAACCTCCATCCGC | 5879 |
| 13025-F3 | GTCAGAAGAAGCACACTGCAG | 4910 |
| 13025-R3 | GAGACTTCATGAATAGGAAGA | 5260 |
| 13025-F4 | GCTGATGACTGGGAGACGAAGC | 3814 |
| 13025-R4 | CCCCTTCCCCGTCTCCTGAGA | 4218 |
| 13025-F5 | GCAGTGCTATGCTGGCTACCCA | 2826 |
| 13025-R5 | TTCAACAGCCCCAAACTCTT | 3668 |
| 13025-F6 | GTCGACCTCTCTCTTTGGGG | 2265 |
| 13025-R6 | TCCTATACTTGAAACCATTATG | 2710 |
| 13025-F7 | GGGTTAATAGGGATGAGGCT | 1469 |
| 13025-R7 | AGGAACGGAGGGCTCAACTT | 1935 |
| LD-prime | CACTGGCGGCCGCTCGAGCATGTAC | 3'end |
| LD-polyT | CACTGGCGGCCGCTCGAGCATGTAC(T)_25_NN | 3'end |

^a^ Positions on the genomes of Aze204, Pair or 13025 agents, as indicated

^b^ In conjunction with LD prime primer

^c^ This reverse primer was used in conjunction with the universal primer provided by the 5’ RACE kit (Takara Bio Europe/Clontech).

^d^ These primers were also used for the amplification of Pair agent.
