# Supplementary material for: Characterization of New Isolates of Apricot vein clearing-associated virus and of a New Prunus-Infecting Virus: Evidence for Recombination as a Driving Force in Betaflexiviridae Evolution
Source: PLoS One. 2015 Jun 18;10(6):e0129469. doi: 10.1371/journal.pone.0129469 (PMC4472227; doi:10.1371/journal.pone.0129469)
Supplement: S2 Table — (DOCX) [file pone.0129469.s005.docx]

**S2 Table. Percentages of identity between various regions of *Apricot vein clearing associated virus* (AVCaV) isolate Pair and corresponding regions of AVCaV reference isolate (NC 023295), AVCaV Iran1, and AVCaV 13025.**

|  | 5'NCR^a^ (78) | Pol^b^ (2,021) | MP^b^ (460) | CP^b^ (221) | NB^b^ (139) | 3'NCR^a^ (152) |
| --- | --- | --- | --- | --- | --- | --- |
| Reference Isolate | 57.7 (73) | 95.9 (1,679) | 98.3 (292) | 99.5 (221) | 99.2 (139) | 98.6 (139) |
| 13025 Isolate | 98.7 (78) | 97.5 (2,021) | 98.2 (460) | 99.5 (221) | 98.4 (139) | 98 (152) |
| Iran1 Isolate^c^ | na^d^ | na^d^ | 96.4 (460) | 98.6 (221) | 95.3 (163) | 97.6 (84) |

^a^ Percentages of nucleotide identity. The size of the 5' and 3' non coding regions are indicated in nucleotide between brackets.

^b^ Percentages of amino acid identity. The size of the proteins are indicated in amino acids between brackets.

^c^ Only the 3,037 nt sequence from the PDO fragment to the 3' end NCR was available

^d^ not applicable

NCR, non coding region; Pol, polymerase; MP, movement protein; CP, coat protein; NB, nucleic acid binding protein
