# Supplementary material for: Characterization of New Isolates of Apricot vein clearing-associated virus and of a New Prunus-Infecting Virus: Evidence for Recombination as a Driving Force in Betaflexiviridae Evolution
Source: PLoS One. 2015 Jun 18;10(6):e0129469. doi: 10.1371/journal.pone.0129469 (PMC4472227; doi:10.1371/journal.pone.0129469)
Supplement: S3 Table — (DOCX) [file pone.0129469.s006.docx]

**S3 Table. List of virus species used for the phylogenetic analyses**

| **Virus name** | **Abbreviation** | **Family** | **Genus** | **Accession number** |
| --- | --- | --- | --- | --- |
| *Caucasus prunus virus* | CPrV | *Betaflexiviridae* |  | KM507061 |
| *Apricot vein clearing associated virus* | AVCaV | *Betaflexiviridae* | unassigned | NC 023295, KM507062-KM507070 |
| *Banana virus X* | BanVX | *Betaflexiviridae* | unassigned | AY710267 |
| *Banana mild mosaic virus* | BanMMV | *Betaflexiviridae* | unassigned | NC 002729 |
| *Sugarcane striate mosaic associated virus* | SCSMV | *Betaflexiviridae* | unassigned | NC 003870 |
| *African oil palm ringspot virus* | AOPRV | *Betaflexiviridae* | unassigned | NC 012519 |
| *Cherry green ring mottle virus* | CGRMV | *Betaflexiviridae* | unassigned | NC 001946 |
| *Cherry necrotic rusty mottle virus* | CNRMV | *Betaflexiviridae* | unassigned | NC 002468 |
| *Cherry twisted leaf associated virus* | CTLaV | *Betaflexiviridae* | unassigned | NC 024449 |
| *Apple stem grooving virus* | ASGV | *Betaflexiviridae* | *Capillovirus* | NC 001749 |
| *Potato virus M* | PVM | *Betaflexiviridae* | *Carlavirus* | NC 001361 |
| *Citrus leaf blotch virus* | CLBV | *Betaflexiviridae* | *Citrivirus* | NC 003877 |
| *Citrus leaf blotch virus* Actinidia isolate | Actinidia citrivirus | *Betaflexiviridae* | *Citrivirus* | JN900477 |
| *Apple stem pitting virus* | ASPV | *Betaflexiviridae* | *Foveavirus* | NC 003462 |
| *Potato virus T* | PVT | *Betaflexiviridae* | *Tepovirus* | NC 011062 |
| *Cherry mottle leaf virus* | ChMLV | *Betaflexiviridae* | *Trichovirus* | NC 002500 |
| *Apple chlorotic leaf spot virus* | ACLSV | *Betaflexiviridae* | *Trichovirus* | NC 001409 |
| *Peach mosaic virus* | PcMV | *Betaflexiviridae* | *Trichovirus* | NC 011552 |
| *Grapevine virus A* | GVA | *Betaflexiviridae* | *Vitivirus* | NC 003604 |
| *Shallot virus X* | ShVX | *Alphaflexiviridae* | *Allexivirus* | NC 003795 |
| *Botrytis virus X* | BotVX | *Alphaflexiviridae* | *Botrexvirus* | NC 005132 |
| *Lolium latent virus* | LoLV | *Alphaflexiviridae* | *Lolavirus* | NC 010434 |
| *Indian citrus ringspot virus* | ICRV | *Alphaflexiviridae* | *Mandarivirus* | NC 003093 |
| *Potato virus X* | PVX | *Alphaflexiviridae* | *Potexvirus* | NC 011620 |
| *Sclerotinia sclerotiorum debilitation-associated RNA virus* | SSDaV | *Alphaflexiviridae* | *Sclerodarnavirus* | NC 007415 |
