# Supplementary material for: Characterization of New Isolates of Apricot vein clearing-associated virus and of a New Prunus-Infecting Virus: Evidence for Recombination as a Driving Force in Betaflexiviridae Evolution
Source: PLoS One. 2015 Jun 18;10(6):e0129469. doi: 10.1371/journal.pone.0129469 (PMC4472227; doi:10.1371/journal.pone.0129469)
Supplement: S4 Table — (DOCX) [file pone.0129469.s007.docx]

**S4 Table.** **List of *Prunus* sources found to be infected by a virus belonging to the proposed genus Prunevirus.**

| **Source** | **Host** | **Country** | **Virus detected** |
| --- | --- | --- | --- |
| Aze204 | *Prunus amygdalus* | Azerbaijan | CPrV^b^ |
| Pair | *Prunus salicina* | France | AVCaV, PPV, PBNSPaV, CVA, PNRSV^b^ |
| 13025 | *Prunus salicina* | France | AVCaV, PBNSPaV^b^ |
| Iran1 | *Prunus persica* | Iran | AVCaV, new *Trichovirus* species^b^ |
| S4 | *Prunus mume* | China | AVCaV, PPV^b^ |
| S15 | *Prunus armeniaca* | China | AVCaV, CVA^b^ |
| 381-07-4 | *Prunus domestica* | France | AVCaV, PNRSV^b^ |

^a^ Complete genome

^b^ The abbreviations of virus names are given in S3 Table.
